# 455. Diagnostic Value of Hematological Parameters in Different Stages of the Disease in Covid-19 Patients

**DOI:** 10.1093/ofid/ofab466.654

**Published:** 2021-12-04

**Authors:** Ruhma Ali, Aditya Patel, Kok Hoe Chan, Jihad Slim

**Affiliations:** 1 Saint Michael’s medical center, Harrsion, New Jersey; 2 Saint Michael’s Medical Center, Newark, New Jersey

## Abstract

**Background:**

COVID-19 infection is associated in some individuals with a rapid onset of systemic proinflammatory state leading to cytokine storm followed by multisystem organ failure. We are interested in studying the prognostic value of complete blood count parameters in different stages of the diseases based on the serology.

**Methods:**

This is a retrospective cohort study of patients with confirmed COVID-19 admitted to our hospital between 10/1/2020 to 2/28/2021. Study individuals had complete CBC profile and COVID-19 serology with well-defined clinical outcome (discharged alive or expired). They were divided in 3 groups based on serology results: group 1 (early disease) had no antibodies, group 2 (immune phase) had + IgM, and group 3 (late phase) had only + IgG. Demographic, clinical and laboratory data were reviewed. Simple t-test was used for continuous variables and chi-square test was used for categorical variables. Anova test was used to compare the difference across multiple groups. GraphPad PRISM was used for all analysis.

**Results:**

A total of 202 confirmed covid 19 cases were included in the study. There was no difference between the 3 groups in terms of age, gender, and body mass index (BMI). We did observe an increase in incidence in Latinx (group 1, 34%; group 2, 51%; group 3, 38%). Hypertension and diabetes were major co-morbidities in these patients. Absolute neutrophil count (ANC) and platelet count (PC) showed significant changes across the 3 groups: mean ANC for group 1, 4.868 (SD 3.117); group 2, mean 6.951 (SD 3.843); and group 3 mean 5.59 (SD 3.236). PC in group 1 mean 193.2 (SD 90.25); group 2 mean 271.1 (SD 143.4); and group 3 mean 228.6 (SD 75.33) p-value 0.0008. The difference can be seen in the derived monocyte platelet rationMPR, neutrophil lymphocyte ratio NLR, platelet lymphocyte ratio PLR and aggregate index of systemic inflammation AISI values and they tend to be higher in group 2 (MPR p-value 0.0067, NLR p-value 0.0123, PLR p-value 0.0294, AISI p-value of 0.0190).

Baseline characteristic

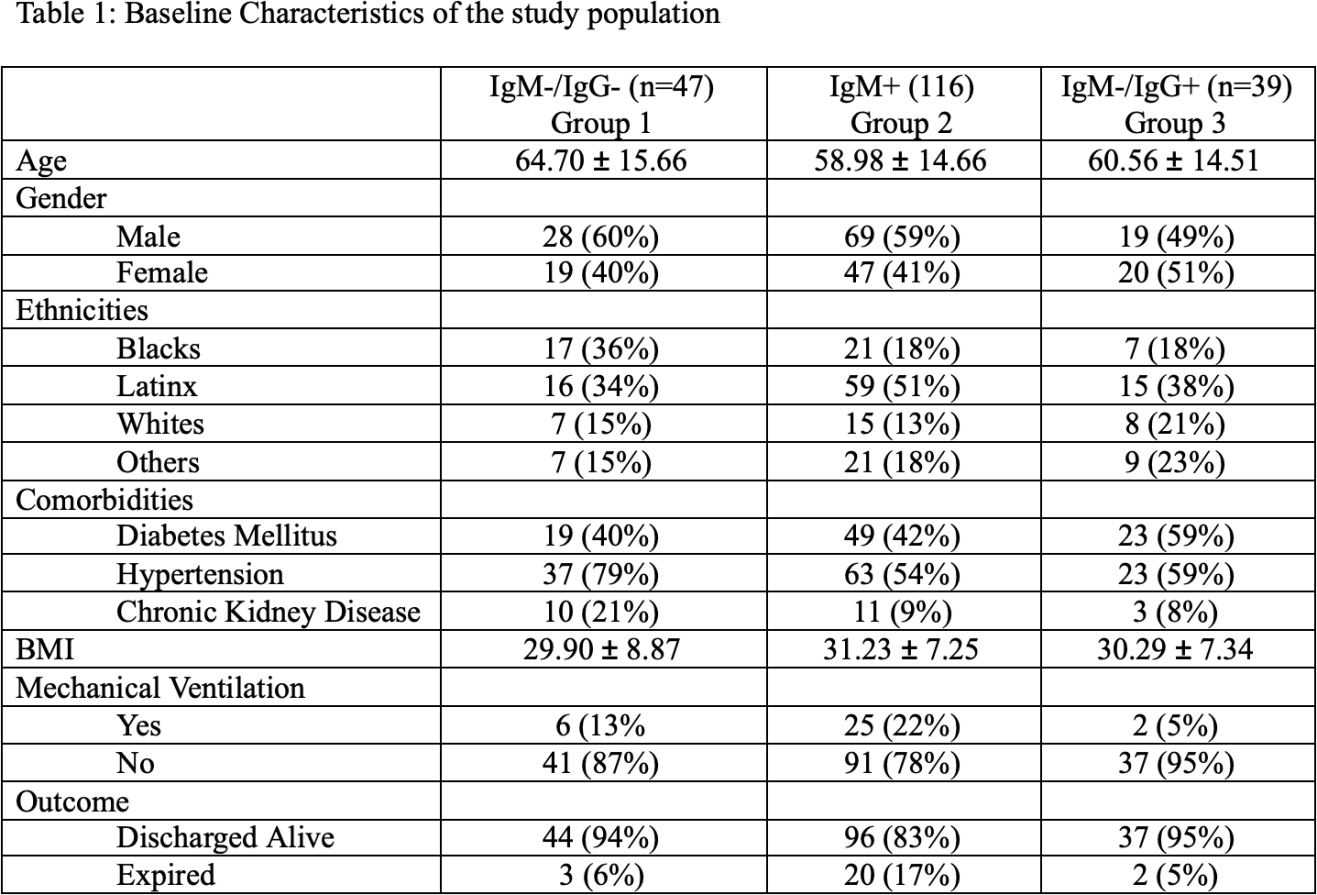

CBC parameters

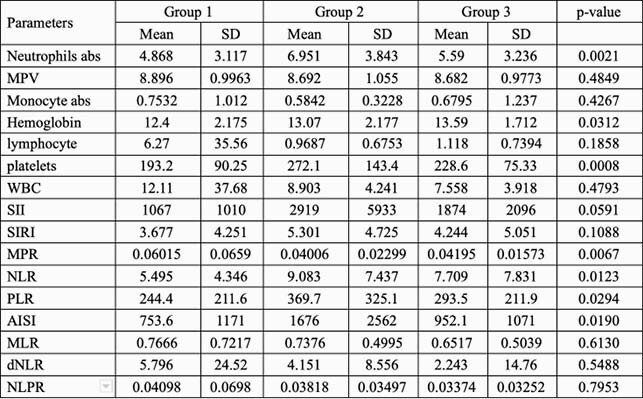

**Conclusion:**

The study demonstrates that MPR, NLR, PLR and AISI have a potential role in categorizing the disease stage based on only CBC profiling.Properly designed prospective studies with a larger sample size should be performed to confirm the disease stratification ability of derived CBC indices like MPR, NLR, PLR and AISI.

**Disclosures:**

**All Authors**: No reported disclosures

